# Acute fibrinous and organizing pneumonia: A report of 13 cases in a tertiary university hospital

**DOI:** 10.1097/MD.0000000000004073

**Published:** 2016-07-08

**Authors:** Rita Gomes, Eva Padrão, Hans Dabó, Filipa Soares Pires, Patrícia Mota, Natália Melo, José Miguel Jesus, Rui Cunha, Susana Guimarães, Conceição Souto Moura, António Morais

**Affiliations:** a Pulmonology Department, Hospital Sousa Martins, ULS-Guarda, Guarda, Portugal; b Faculty of Health Sciences, University of Beira Interior, Covilhã, Portugal; c Pulmonology Department, Centro Hospitalar de São João, Porto, Portugal; d Faculty of Medicine, University of Porto, Portugal; e Radiology Department, Centro Hospitalar de São João, Porto, Portugal; f Pathology Department, Centro Hospitalar de São João, Porto, Portugal.

**Keywords:** acute fibrinous and organizing pneumonia (AFOP), etiology, treatment

## Abstract

**Introduction:**

Acute fibrinous and organizing pneumonia (AFOP) is a rare diffuse pulmonary disease, but it is not yet known whether it is a distinct form of interstitial pneumonia or simply a reflection of a tissue sampling issue.

**Methods:**

Cross-sectional evaluation of clinical and radiological findings, treatments, and outcomes for patients with histologically confirmed AFOP at a tertiary university hospital between 2002 and 2015.

**Results:**

Thirteen patients (7 women, 53.8%) with a mean ± SD age of 53.5 ± 16.1 years were included. The main symptoms were fever (69.2%), cough (46.2%), and chest pain (30.8%). All patients presented a radiological pattern of consolidation and 5 (38.5%) had simultaneous ground-glass areas. Histology was obtained by computed tomography-guided transthoracic biopsy in 61.5% of cases and by surgical lung biopsy in the remaining cases. Several potential etiologic factors were identified. Eight patients (61.5%) had hematologic disorders and 3 had undergone an autologous hematopoietic cell transplant. Two (15.4%) had microbiologic isolates, 5 (38.4%) had drug-induced lung toxicity, and 2 (15.4%) were classified as having idiopathic AFOP. In addition to antibiotics and diuretics used to treat the underlying disease, the main treatment was corticosteroids, combined in some cases with immunosuppressants. Median survival was 78 months and 6 patients (46.2%) were still alive at the time of analysis.

**Conclusion:**

Our findings for this series of patients confirm that AFOP is a nonspecific reaction to various agents with a heterogeneous clinical presentation and clinical course that seems to be influenced mainly by the severity of the underlying disorder.

## 1 Introduction

Acute fibrinous and organizing pneumonia (AFOP) is a histological pattern characterized predominantly by the presence of intra-alveolar fibrin in the form of fibrin “balls” within the alveolar spaces, with a patchy distribution, and organizing pneumonia.^[1,2]^ It was first described in 2002 by Beasley et al^[1]^ in a series of 17 patients. Since then, there have been essentially isolated reports describing diverse causes and clinical courses.^[3–6]^ Consequently, whether AFOP is a distinct pattern of interstitial pneumonia or whether it simply reflects a tissue sampling issue remains to be elucidated.^[2]^

A variety of causes have been linked to AFOP, including infections, drugs, immune status, and occupational exposures, but idiopathic cases have also been described. The condition frequently occurs in the context of an underlying disease (Table 1).^[1,4,7–12]^

**Table 1:**
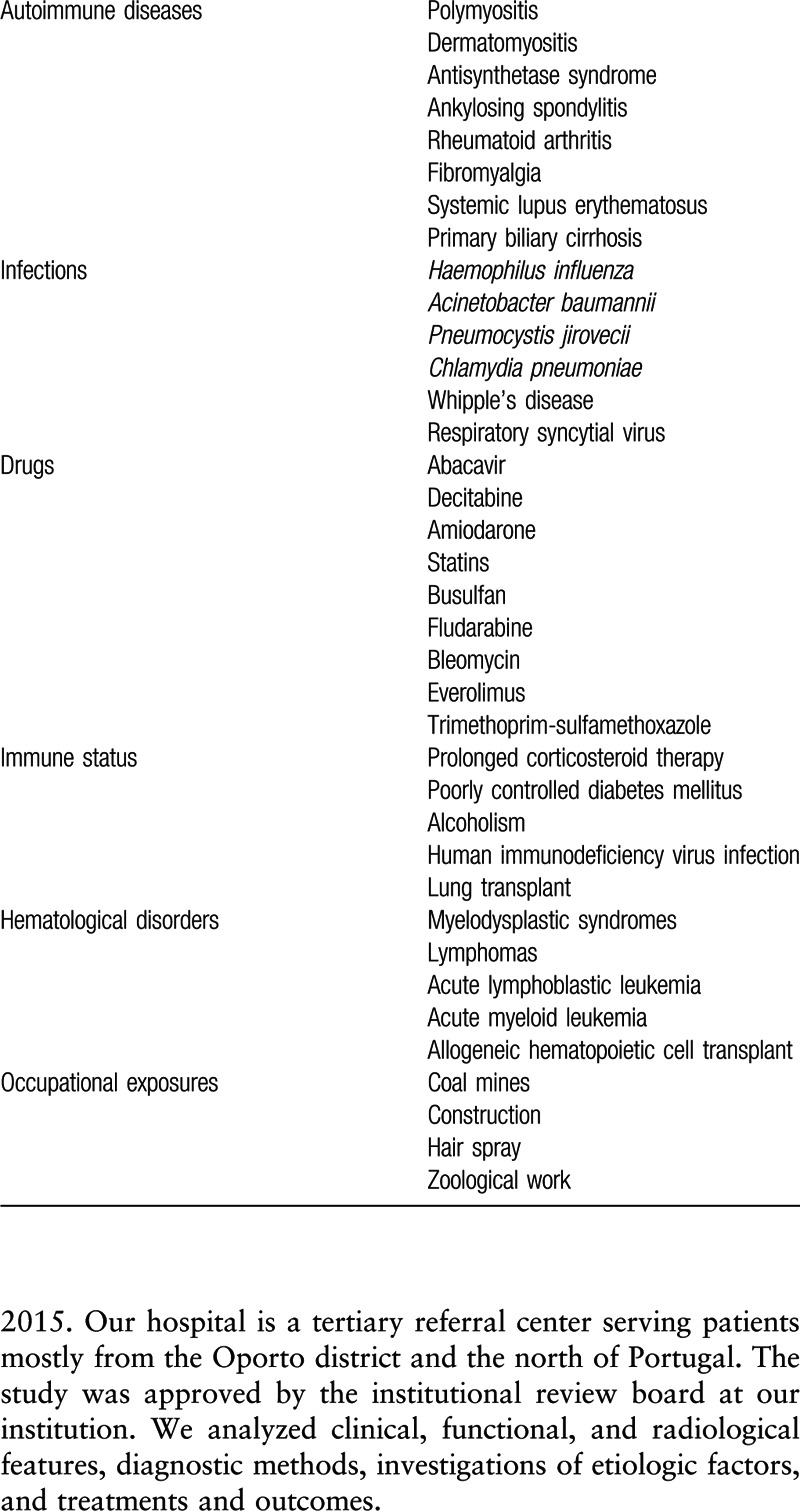
Possible etiologic and risk factors in acute fibrinous and organizing pneumonia.

Given the variability in clinical, radiological, and bronchoalveolar lavage findings, diagnosis requires the detection of characteristic features of AFOP in a lung tissue sample.^[1,2,13–15]^ Numerous treatments have been reported for AFOP, but considering the rarity of the condition, together with the diversity of clinical presentations and underlying conditions, there are no standard treatment recommendations.^[1,3,5,16–19]^ Data on outcomes also vary due to this diversity, but a significant number of cases have been associated with poor prognosis.^[1,18]^ The real influence of AFOP versus its causes or associated conditions remains to be elucidated.

The aim of the study was to describe the clinical evaluation and course of patients with a histological diagnosis of AFOP in a tertiary hospital, to report on the treatments prescribed, and to explore prognostic factors associated with different outcomes.

## 2 Methods

### 2.1 Study design

We performed a cross-sectional study of patients with a histological diagnosis of AFOP evaluated at the Centro Hospitalar São João in Oporto, Portugal, between 2002 and 2015. Our hospital is a tertiary referral center serving patients mostly from the Oporto district and the north of Portugal. The study was approved by the institutional review board at our institution. We analyzed clinical, functional, and radiological features, diagnostic methods, investigations of etiologic factors, and treatments and outcomes.

**Figure 1:**
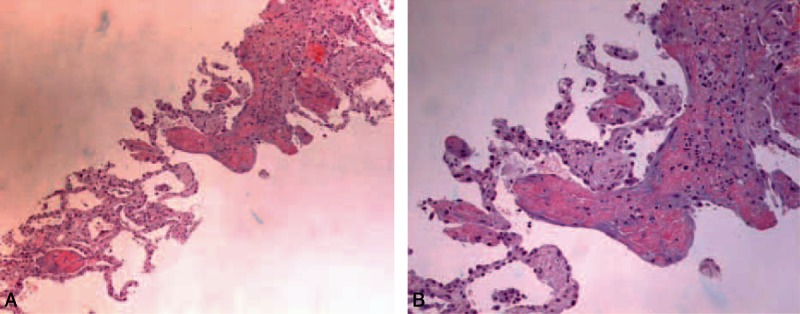
Histologic findings of acute fibrinous and organizing pneumonia: (A) hematoxylin and eosin stain, ×100; (B) hematoxylin and eosin stain, ×200. Alveoli containing fibrin “balls” and some granulation tissue, in a patchy distribution.

The histological criteria used were those defined in 2002 by Beasley et al^[1]^ that is, the presence of intra-alveolar fibrin in the form of fibrin balls within the alveolar spaces in a patchy distribution and organizing pneumonia consisting of intraluminal loose connective tissue within bronchioles and alveolar ducts. Additional features described include hyperplasia of type II pneumocytes, alveolar septal expansion, and acute and/or chronic inflammation (Fig. 1).

### 2.2 Statistical analysis

We used the Kolmogorov–Smirnov test (*P ≥* 0.05) to determine the normality of continuous variables and Levene's test to assess equality of variances. Differences between means were analyzed using the *t* test for normally distributed variables. A Kaplan–Meier curve was used for the survival analysis. *P* values < 0.05 were considered to be statistically significant. Statistical analyses were performed using IBM SPSS Statistics for Windows, version 19.0.

## 3 Results

We analyzed 13 patients with a histological diagnosis of AFOP treated at our hospital between 2002 and 2015. Seven (53.8%) were women and 6 (46.2%) were men. The mean ± SD age (Kolmogorov–Smirnov test, *P* = 0.956) was 53.5 ± 16.1 years, and was higher in men (60.8 ± 16.2) than in women (47.1 ± 14.1), although the difference was not statistically significant (*P* = 0.131). Mean time from onset of symptoms to diagnosis was 43.9 ± 33.0 days. The clinical presentation included fever in 9 patients (69.2%), cough in 6 (46.2%), chest pain in 4 (30.8%), constitutional symptoms in 3 (23.1%), and dyspnea in 2 (15.4%). The presentation was acute in 2 patients (15.4%) and subacute in the remaining 11 (84.6%). One patient (7.7%) was asymptomatic.

**Figure 2:**
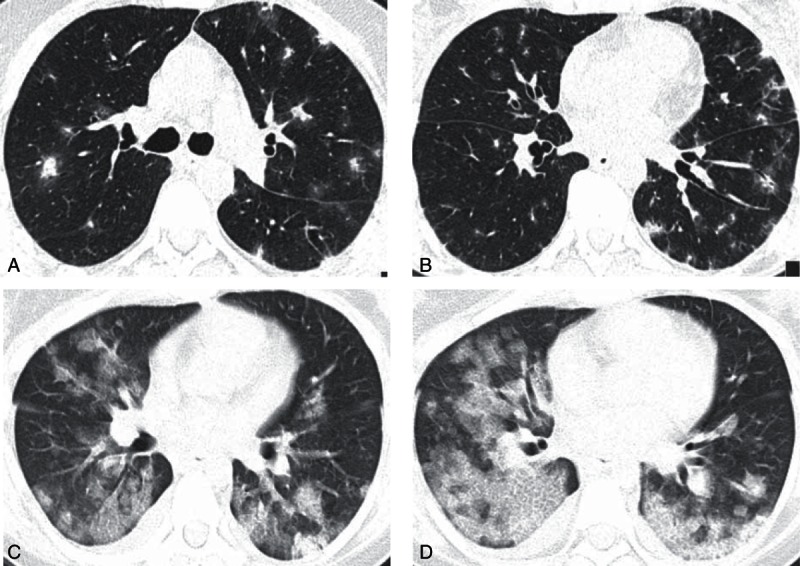
Radiological findings of acute fibrinous and organizing pneumonia. (A, B) High-resolution computed tomography images showing focal areas of airspace consolidation and ground glass along the bronchovascular bundles, with a diffuse random distribution. (C, D) Computed tomography images showing diffuse areas of airspace consolidation and ground glass along the bronchovascular bundles.

All patients presented a radiological pattern of consolidation, and 5 (38.5%) had a consolidation pattern together with ground-glass areas in the high-resolution computed tomography chest scan; the distribution was mostly diffuse random, but 3 patients (23.1%) showed a diffuse peribronchovascular distribution (Fig. 2). AFOP histology was obtained by computed tomography-guided lung transthoracic biopsy in 8 patients (61.5%) and by surgical lung biopsy in 5 (38.5%).

The majority of patients (n = 8, 61.5%) had hematologic disorders: 4 (30.4%) had lymphomas (Hodgkin in 3 cases and non-Hodgkin in 1), 2 (15.4%) had acute leukemia (acute lymphoblastic leukemia and acute myeloid leukemia), 1 (7.7%) had myelodysplastic syndrome, and 1 (7.7%) had multiple myeloma. Three patients (23.1%) with lymphoma had undergone an autologous hematopoietic cell transplant. Two of the patients (15.4%) also had microbiologic isolates, specifically *Acinetobacter baumannii* and *A fumigatus.* All 8 patients were receiving medication for their hematologic disorders. However, of all the drugs evaluated, only azacytidine and bleomycin have been linked to AFOP to date.^[17,20]^

The patient with multiple myeloma was being treated with everolimus and prolonged corticosteroid therapy for a kidney transplant performed 7 years earlier.

Another patient (7.7%) had undergone a kidney transplant 6 years earlier and was being treated with sirolimus. Although this drug has never been associated with AFOP, sirolimus can induce a range of adverse respiratory events, and other immunosuppressive macrolides, such as everolimus, have been linked to AFOP.^[21,22]^ In this case, thus, sirolimus was identified as the probable cause of AFOP. One patient (7.7%) had breast cancer and had undergone surgery followed by adjuvant chemotherapy (docetaxel) and radiotherapy. Although neither of these therapies has been linked to AFOP, the co-occurrence of AFOP means that this association must be considered. Two patients (15.4%) were considered to have idiopathic AFOP after an extensive search for possible causes. One patient (7.7%) had AFOP only on the periphery of a lung abscess, and it was considered to be simply a histological feature of this lesion.

Treatment included antibiotics in 11 patients (84.6%), corticosteroids in 10 (76.9%), mycophenolate mofetil in 2 (15.4%), and cyclophosphamide in 2 (15.4%). Drugs suspected to be associated with AFOP were withdrawn. Three patients (23.1%) required invasive mechanical ventilation (IMV) in an intensive care unit.

The median survival according to the Kaplan–Meier curve was ∼78 months, and 6 patients (46.2%) were still alive at the time of our analysis. The mean duration of follow-up for these 6 patients was 76.7 ± 54.6 months; 5 (83.3%) had acute disease, and 1 (16.7%) developed chronic disease. Seven patients (53.8%) died. AFOP was the cause of death in 3 cases (42.9%); the other 4 patients (57.1%) died of other causes: lymphoma/leukemia progression in 2 cases and septic/hemorrhagic shock in the other 2. The mean time from the onset of symptoms to death due to AFOP progression was 89.3 ± 104.5 days.

Table 2 summarizes the clinical characteristics and treatments administered for the 13 patients.

**Table 2:**
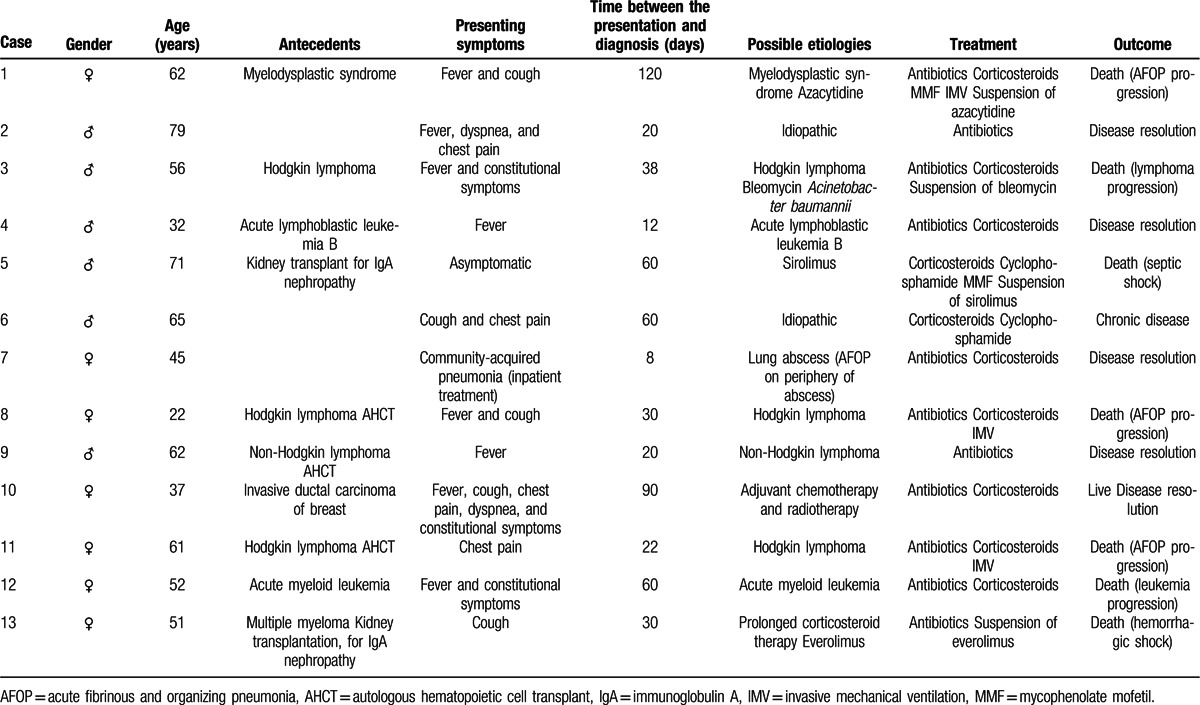
Clinical characteristics and treatment of AFOP patients.

## 4 Discussion

Our identification of just 13 cases of histologically confirmed AFOP in a tertiary referral hospital over a period of 14 years confirms the rarity of this entity. The age of the patients was diverse and there was no clear gender difference. A significant number of patients (n = 8) had an underlying hematologic disorder, but the majority of patients had several potential causes of AFOP, including drugs and infectious agents. Due to the diversity of conditions and, consequently, treatments, we were unable to identify any specific potentially effective drugs. Clinical outcomes were also diverse, and ranged from resolution to death (53.8% of cases). We were unable to identify prognostic factors due to the small sample size and the significant number of comorbidities and complications during the clinical course of the patients who died.

AFOP is a rare histological pattern of acute lung injury. It is a relatively new entity that requires better clinical and radiologic characterization and the identification of poor outcome markers. Numerous causes have been associated with AFOP to date.^[1,4,7–12]^ In our sample, 8 patients (61.5%) had a hematologic disorder. AFOP has been linked to hematologic disorders in isolated reports and also in the only 2 cases series published to date, although to a considerably lower extend than in our series.^[1,6,7,23,24]^ Our institution is a university hospital with 1100 beds and covers all medical and surgical specialities, with no particular predominance. One possible explanation for the preponderance of hematologic disorders is that these patients may need aggressive chemotherapy, leading to immunosuppression and consequently the risk of opportunistic infections, which are all possible causes of AFOP. We identified several potential etiologic factors, mostly related to infections (*A baumannii* and *A fumigatus)* or drug-induced toxicity (azacytidine, bleomycin, everolimus, and sirolimus), in a significant number of cases. To our knowledge, this is the first series of AFOP to report sirolimus as a potential cause of AFOP, and its implication is based on previous reports of an association between AFOP and drugs with similar pharmacologic proprieties.^[21,22]^ We also believe that combined chemoradiotherapy may have been responsible for AFOP in 1 patient because of the co-occurrence in time and the absence of other probable causes. Although there have been reports of idiopathic AFOP,^[5,19,25]^ an exhaustive investigation is critical as etiology seems to be an important determinant of prognosis and consequently of treatment.

The presenting symptoms of AFOP were nonspecific. In our sample, the mean time from onset of symptoms to diagnosis was 43.9 days, which is significantly longer than the 19 days described by Beasley et al.^[1]^ Two patients had symptoms for >60 days. This considerable diagnostic delay may be due to the nonspecific nature of the presenting symptoms, which in many cases overlapped with those of several of the comorbidities observed. AFOP has no pathognomonic or specific radiologic features, although bilateral basal opacities are frequently seen, as are occasional diffuse areas of consolidation, sometimes with bronchovascular bundles.^[1,14]^

AFOP has no specific treatment and therapeutic strategies vary considerably according to the underlying disease and clinical presentation.^[1,3,5,16–19]^ Coinciding with reports in the literature, the treatments used in our series were diverse. In addition to specific treatments for the underlying disease, such as antibiotics or diuretics, AFOP treatments included mostly steroids, combined in some cases with immunosuppressants, such as cyclophosphamide or mycophenolate mofetil. However, none of the treatments was identified as being particularly beneficial.

In the series described by Beasley et al,^[1]^ 30% of patients required IMV and they all died. In our sample, IMV was used in 23.1% of patients, who all died due to progression of AFOP. This observation would appear to confirm Beasley et al's^[1]^ suggestion that there are 2 main forms of disease: a fulminant form leading to rapid deterioration and death and another, subacute form, which in some cases may resolve after treatment with corticosteroids. Median survival in our sample was ∼78 months. The mortality rate described in the literature is >50%,^[1]^ which coincides with our findings, as 7 (54%) of the patients in our series died. Three of the deaths were due to AFOP progression. Because of the small number of patients, we were unable to isolate any prognostic factors. However, the fact that the 2 patients considered to have idiopathic AFOP had longer survival times deserves some attention, as it could be speculated that it is largely the underlying condition and not AFOP itself that is associated with poor prognosis.

In conclusion, this cohort analysis confirmed that AFOP is a nonspecific reaction to various agents that follows a heterogeneous clinical course, with variable presentations, that seems to be largely influenced by the severity of the underlying disorder.
